# Nuclei Detection and Segmentation of Histopathological Images Using a Feature Pyramidal Network Variant of a Mask R-CNN

**DOI:** 10.3390/bioengineering11100994

**Published:** 2024-10-01

**Authors:** Vignesh Ramakrishnan, Annalena Artinger, Laura Alexandra Daza Barragan, Jimmy Daza, Lina Winter, Tanja Niedermair, Timo Itzel, Pablo Arbelaez, Andreas Teufel, Cristina L. Cotarelo, Christoph Brochhausen

**Affiliations:** 1 Institute of Pathology, University Regensburg, Franz-Josef-Strauß-Allee 11, 93053 Regensburg, Germany; 2 Central Biobank Regensburg, University and University Hospital Regensburg, Franz-Josef-Strauß-Allee 11, 93053 Regensburg, Germany; 3 Institute of Pathology, Medical Faculty Mannheim, Heidelberg University, Theodor-Kutzer-Ufer 1-3, 68167 Mannheim, Germany; 4 Center for Research and Formation in Artificial Intelligence (CinfonIA), Universidad de Los Andes, Cra. 1 E No. 19A-40, Bogotá 111711, Colombia; 5 Department of Internal Medicine II, Division of Hepatology, Medical Faculty Mannheim, Theodor-Kutzer-Ufer 1-3, 68167 Mannheim, Germany; 6 Clinical Cooperation Unit Healthy Metabolism, Center for Preventive Medicine and Digital Health, Medical Faculty Mannheim, Heidelberg University, 69117 Mannheim, Germany

**Keywords:** digital pathology, Mask R-CNN, nuclei detection, artificial intelligence, histopathology

## Abstract

Cell nuclei interpretation is crucial in pathological diagnostics, especially in tumor specimens. A critical step in computational pathology is to detect and analyze individual nuclear properties using segmentation algorithms. Conventionally, a semantic segmentation network is used, where individual nuclear properties are derived after post-processing a segmentation mask. In this study, we focus on showing that an object-detection-based instance segmentation network, the Mask R-CNN, after integrating it with a Feature Pyramidal Network (FPN), gives mature and reliable results for nuclei detection without the need for additional post-processing. The results were analyzed using the Kumar dataset, a public dataset with over 20,000 nuclei annotations from various organs. The dice score of the baseline Mask R-CNN improved from 76% to 83% after integration with an FPN. This was comparable with the 82.6% dice score achieved by modern semantic-segmentation-based networks. Thus, evidence is provided that an end-to-end trainable detection-based instance segmentation algorithm with minimal post-processing steps can reliably be used for the detection and analysis of individual nuclear properties. This represents a relevant task for research and diagnosis in digital pathology, which can improve the automated analysis of histopathological images.

## 1. Introduction

The detection and the interpretation of cell nuclei represent crucial issues in histopathological diagnostics. Pathologists have to examine nuclear features, such as numbers, pleomorphisms, nuclear-to-cytoplasmic ratios, or morphologic irregularities, not only to differentiate benign from malignant cells but also to grade malignant tumors, with relevant consequences for staging and further treatment options [1,2]. Therefore, analyzing nuclei in histopathological tissues is an integral part of diagnostic examinations or research in pathology [3]. Due to the fast development of artificial intelligence (AI), computational pathology is trending, with new algorithms and improved scanners for whole-slide imaging (WSI). Research to diagnose and provide appropriate therapy—for example, for cancer or any other disease—can now be supported with the help of AI [4]. Given the success of convolutional neural networks (CNNs), deep-learning-based approaches have been proposed for the automated analysis of histopathological images [5].

Various approaches for nuclei-based image analyses exist already. One class of algorithms is semantic segmentation algorithms, which focus on pixel-accurate results from images. It can be used in pathological analyses to compare attributes like the area and edges of nuclei in a particular region and to isolate the nuclear boundaries of tissue [6]. However, the individual properties of each nucleus and the exact location cannot be determined without post-processing techniques. Since Otsu’s thresholding method in 1979 [7], semantic segmentation algorithms have come a long way [8]. The most recent algorithms making the headlines today are based on deep learning using architectures like UNet [9]. A comprehensive survey on semantic segmentation algorithms is provided in an elaborate way elsewhere [10,11].

Object detection algorithms, on the other hand, are a different class of algorithms that focus only on locating and counting nuclei for pathological applications [12]. The nuclear boundaries and their individual area cannot be determined. Compared with semantic segmentation, object detection has evolved only in the last 20 years [13], from a simple Viola–Jones detector [14] to a deep-learning-based single-stage YOLO detector [15] or a two-stage Faster R-CNN detector [16]. In general, the historical evolution of object detection algorithms is an interesting topic that has been extensively reviewed in the literature [17,18].

Both semantic segmentation and object detection are necessary for the complete analysis of tissue, giving rise to a new class of algorithms: instance segmentation [19]. Instance segmentation algorithms are widely used for several applications, including nuclei instance segmentation, cell segmentation, and mitosis detection [3]. Ho et al. used a nuclei detection and segmentation algorithm on fluorescence images to study rat kidneys [20]. These approaches also provided an innovative input into research involving single-nucleus RNA sequencing in kidney fibrosis [21] and a multi-omic single-nucleus study on late-stage Alzheimer’s disease [22]. More generally, automated nucleus detection and consecutive characterization represent innovative approaches for cell counting, not only in tissue slides but also in in vitro cell constructs, such as spheroids or organoids. Furthermore, the detection of nuclei gives the base for further steps, namely, the interpretation of the nucleus structure with a view to malignancy. In the future, these techniques can be integrated into daily pathological routines, facilitating cell nuclear interpretation and reducing potential individual bias.

Instance segmentation approaches can be classified into semantic-segmentation-based instance segmentation (SIS) and detection-based instance segmentation (DIS). SIS uses traditional post-processing techniques like a marker-based watershed algorithm to separate the contours of nuclei [23] or to introduce a contour-aware network to identify the contours of nuclei [24]. However, DIS directly uses object-detection-based methods to predict segmented masks for each object, for example, with the help of a class-agnostic segmentation branch [25]. The following table (Table 1) summarizes previous studies and their methodologies.

A survey on deep learning in digital pathology by Deng et al. [3] shows that most medical applications use SIS algorithms, indicating significant research in SIS as compared to DIS. SISs such as CNN or U-Net [27,29] may be simple and fast, but they exhibit heavily engineered post-processing techniques. Over-segmentation leads to additional nuclei and under-segmentation fails to separate crowded nuclei. This implies that the nuclei boundaries cannot be managed correctly, making SIS difficult to generalize. U-Net is an example of a Fully Convolutional Network (FCN). FCNs can become very complex, especially when deep architectures are used. This complexity can lead to challenges in training, such as overfitting or the vanishing gradient problem, which might require extensive tuning and regularization. Also, FCNs are designed for semantic segmentation and can have difficulties with other tasks like object detection or instance segmentation. Some detection-based methods [25,30], while effective, require substantial computational resources, making them less accessible for real-time clinical applications or use in resource-limited settings. Hybrid models like Hover-Net, which combine aspects of SIS and DIS, also face similar challenges. In contrast, since DIS inherently focuses on detection, it has end-to-end trainable solutions with minimal post-processing techniques and exhibits high performance. Therefore, it is easier to generalize, although it is time-consuming and includes certain false positives and false negatives.

One way to evaluate the persuasiveness of the current algorithms for nuclei segmentation is by running the networks on the Kumar Dataset, a public dataset containing hematoxylin and eosin (H&E)-stained tissue images from seven different organs [27]. Based on the available results on Kumar Dataset [26], it is evident that SIS, like HoVer-Net [30] and DSF-CNN [23], give a much higher accuracy of 82.6% each on the dice score of nuclei segmentation as compared to a DIS like Mask R-CNN [25], which gives a 76% dice score.

We consider the following points in our work:

- SIS-based approach vs. DIS-based approach;
- Limitation of SIS-based approach: use of traditional post-processing algorithms;
- Use of DIS-based approach for nuclei detection: end-to-end trainable and no need for traditional post-processing algorithms to detect nuclei.

Therefore, in the present study, we attempt to increase the dice score accuracy of a DIS network, the Mask R-CNN by enhancing its architecture using a variant based on Feature Pyramidal Networks (FPN) [31] and adapting the framework to use some of the modern training setups. The intention of integrating an FPN variant is to accommodate nuclei of various scales and sizes in different organs. Since FPN networks are used to exploit multi-scale, pyramidal representations of deep convolutional neural networks to consider objects of various scales [31], they are used for this research. The algorithm minimizes reliance on traditional post-processing techniques, thereby reducing the risk of over-segmentation and improving generalization across diverse datasets. Furthermore, the proposed approach is designed to be computationally efficient, enabling real-time application in clinical workflows without sacrificing accuracy. By leveraging a more diverse dataset that includes a wide range of tissue types and pathological conditions, this study also aims to enhance the robustness and applicability of the model across various histopathological scenarios.

Firstly, the methods and metrics used in our study are described. These include the network that represents the baseline, the dataset used for training, the training of the network, the tuning of the parameters, and the metrics used to measure efficiency. In the results section, the efficiency of the algorithm is emphasized and compared with similar approaches and presented using images. The reasons for our approaches, as well as the points to be considered in future studies, are set out in the discussion. At the end of this paper, our work is summarized once again in the conclusion.

## 2. Materials and Methods

### 2.1. Network

**Figure 1:**
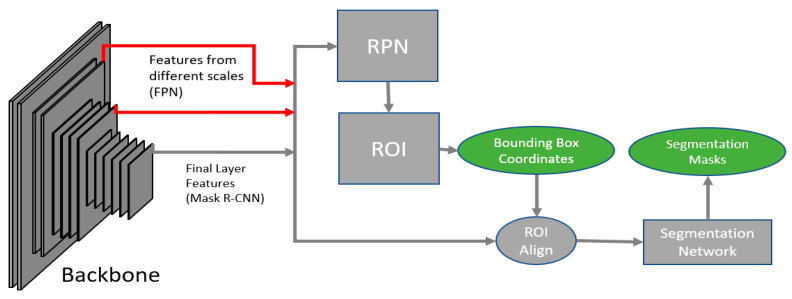
Mask R-CNN architecture showing FPN modification. The red arrows depict the changes in the FPN variant of the Mask R-CNN.

Mask R-CNN comprises two main stages (Figure 1), excluding the backbone network. The input image is first passed through a backbone network like the ResNet50 to result in intermediate feature layers. The features are provided as inputs to the two main stages of a Mask R-CNN. The first stage, known as the Region Proposal Network (RPN), focuses on localizing objects using anchors, which are a set of bounding boxes defined at every pixel of the input image. The second stage is a Region of Interest (ROI) stage, which comprises a classifier to predict the class of the object and a regressor network to adjust the boundary position of the bounding box. An additional stage is introduced in the end, to semantically segment the object from the resulting bounding boxes.

Anchors are defined as a list of anchor sizes and their corresponding anchor scales. Anchors are chosen such that the features from the final layer of a backbone network are provided as inputs to the RPN stage in a simple Mask R-CNN network. In the FPN variant, various sets of anchors are chosen for different feature layers of the backbone network and each of these feature layers is passed on to the RPN stage. The RPN stage comprises a classifier and a regressor network. The classifier network filters out all anchors containing an object and the regressor network adjusts the anchor box size. Non-Maximum Suppression (NMS) [32] is used to eliminate duplicate bounding boxes. This results in a list of bounding box proposals, which are passed as inputs to the ROI stage (Figure 1).

The ROI stage comprises another classifier network to identify the class of the proposed bounding box and a regressor network to fine-tune the bounding box. The irrelevant bounding boxes from the given set of proposals are removed in this stage. For our use case, the bounding boxes containing nuclei are our outputs. Each nucleus contains the coordinates of its bounding box and the relevant class.

Mask R-CNN is an instance segmentation algorithm. This means that the object is not only detected as a bounding box, but it is also semantically segmented. Therefore, an additional semantic segmentation network is trained on the resulting bounding box nuclei. An ROI pooling algorithm is used to convert the uneven bounding boxes to a constant image size, which is passed through a semantic segmentation network, to observe the boundaries of the object within the bounding box.

The specifics of the architecture used in this study are as follows: A ResNet50 [33] is used as its backbone network. It was initialized with pre-trained weights [34] on the ImageNet dataset [35]. Anchor sizes are chosen separately for each feature scale such that the features with higher scales use lower anchor sizes and vice versa. The anchors are classified as positive if the Intersection over Union (IoU) is above 0.4 and negative if it is below 0.1. The classifier and regressor for the RPN comprise 3 × 3 and 1 × 1 hidden convolution layers. Two fully connected layers are used for the box head and regressor for the ROI stage and four convolution layers for the segmentation head. ROIAlign [25] is adopted as the ROI pooler to interpolate the proposals and generate image inputs of 14 × 14 pixels for the segmentation network.

### 2.2. Dataset

The public dataset used to train and test the Mask R-CNN with FPN is the Kumar dataset [27]. The training data contains a total of 30 histological images of 1000 × 1000 pixels from 7 organs and around 22,000 nuclear boundary annotations. The images comprise 6 breast, 6 kidney, 6 liver, 6 prostate, 2 bladder, 2 colon and 2 stomach images. The validation sets contain 14 images and around 8250 nuclear boundaries. The images are extracted from H&E-stained tissue images captured at 40× magnification and include both benign and diseased tissue samples.

For our experiments, each 1000 × 1000 pixel image is divided into patches of smaller size, adapting the relevant annotations to that patch, e.g., using a patch size of 250 and a stride of 250 would result in a total of 16 patches. In the present study, a patch size of 224 × 224 pixels was used for most of the experiments.

### 2.3. Training

The base framework used for training is Detectron2 [34] for instance segmentation. The dataset mapper is adapted to use patched images from the Kumar dataset. Stochastic Gradient Descent [36] is generally used for object detection due to increased stability and better generalized results. The framework is adapted to use the Adam optimizer [37] for faster learning. A learning rate of 0.001, a momentum of 0.9, and a weight decay factor of 0.0001 is used in most of our experiments. The anchors were chosen to be of sizes [4, 8, 12], [12, 16, 32], [32, 48, 64], [48, 64, 96], and scales [0.5, 1.0, 2], which tells us the anchor sizes and scales corresponding to four intermediate layers of the ResNet50, which are provided as inputs to the RPN stage. The RPN network has Intersection-over-Union (IOU) values of 0.1 and 0.4 to filter out negative and positive bounding boxes, respectively. The RPN and ROI networks used an NMS threshold value of 0.5.

To account for rotation invariance, the images are randomly rotated, and the patches are cropped from the rotated images. To account for changes in contrast, saturation, and brightness, the patches were blended using blending filters. The percentage of the relevant augmentations is chosen such that the change does not exceed 40% of the original image.

There are five main losses to be minimized during training. The classification networks, used in the RPN and ROI stages, use cross-entropy loss. The regression networks in RPN and ROI stages use smooth-L1 loss [38]. The segmentation network uses cross-entropy loss. For some of our experiments, the cross-entropy loss of the RPN stage is changed to focal loss [39] to account for class imbalance while filtering relevant anchors in the RPN stage. In the ROI stage, a percentage of negative proposals are randomly chosen for training. Online hard example mining (OHEM) [40], an approach where the ROI network is forced to choose the negative proposals with relatively high positive scores to minimize false positives, is also used.

### 2.4. Tuning

Tuning was carried out by observing training and validation curves using various methods. A set of values was chosen for each of the relevant parameters and the network was trained multiple times independently. Many parameters were considered to tune, for example, the training rate was adapted to ensure convergence, different loss functions were evaluated, and IoU for RPN, NMS for ROI, etc., were modified for the nuclei sizes.

Also, the default anchor sizes and ratios need to be adapted to detect nuclei, because they were trained to detect other structures like cats and dogs. These structures are much bigger than the nuclei to be detected. Therefore, the average nuclei dimensions are calculated using a training dataset, and multiple combinations of anchor sizes and ratios were evaluated.

### 2.5. Metrics

The metric to measure the reliability of the segmentation results is the Sorensen–Dice Coefficient (‘dice score’) [41]. A dice value of 100 (in percentage) means that the predicted mask is exactly like the target mask. The definition of dice score is given by the following formula, where *X* and *Y* are the two masks to be compared:

$$Dice\left(X,Y\right)\left(\%\right)=\frac{2|X\cap Y|}{\left|X+Y\right|}\times 100$$

**Figure 2:**
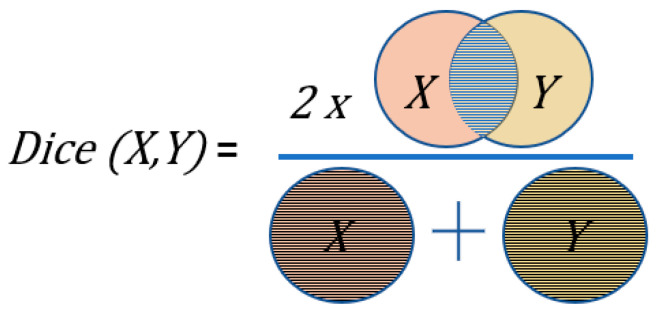
Visualization of the dice score. *X*: prediction (orange), *Y*: ground truth (yellow).

Visually, the dice score can be explained as shown in Figure 2. Let us assume that *X* and *Y* represent the prediction and ground truth of the nuclei masks. The numerator is the overlapped area of *X* and *Y*. The denominator is the sum of the area of *X* and *Y*. The ratio of twice the overlap to the sum of both masks gives the dice score. As an example, if the ground truth and prediction do not overlap, the dice score is 0. Similarly, if they fully overlap, the dice score is 1.

### 2.6. Inference 

Dice score needs to be computed on images with a resolution of 1000 × 1000 pixels, while our network works on patches of 224 × 224 pixels. Therefore, the dice score is evaluated for overlapped patches in the images, and duplicate nuclei instances are filtered at the boundaries using Non-Maximum Suppression (NMS) [28]. As a result, discrepancies in nuclei detection are avoided at the boundaries of patches.

## 3. Results

**Figure 3:**
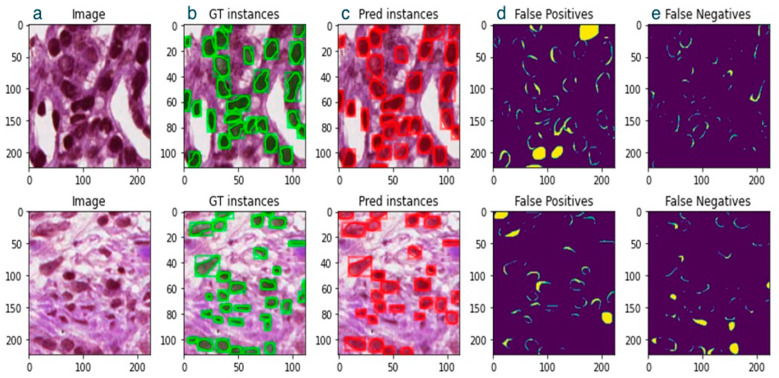
Two example analyses of a patch, (left to right). (**a**) Image, (**b**) ground truth instances (green bounding box + mask), (**c**) predicted instances (red bounding box + mask), (**d**) false positives, (**e**) false negatives.

Due to modern scanners and microscopy systems, histological images have a huge size, and fully zoomed images are of tremendously higher resolution with tens of thousands of nuclei. As for the Kumar dataset, each image has a resolution of 1000 × 1000 pixels, containing hundreds of nuclei [27]. Since the nuclei sizes are significantly small in such large images, it would be necessary to perform a dense analysis of the samples by checking for positive anchors in almost every pixel to detect nuclei if we directly use a large image for DIS networks. Moreover, we would need much deeper networks, and training would require larger datasets and extensive computational resources, resulting in increased complexity and training time of the network. Furthermore, for exceedingly small patches, it is difficult to choose a reasonable overlap between the patches to reconstruct the full image during inference. In the worst case, the object itself might be too big for the patch size. Consequently, since nuclei sizes range from 8 × 8 to 80 × 80 pixels, a reasonable patch size of 224 × 224 pixels was chosen as input to the Mask R-CNN (Figure 3). The improvement in dice score accuracy on the FPN variant of Mask R-CNN was evident after a step-by-step analysis of various training setups (Table 2).

The baseline Mask R-CNN gives an accuracy of 76.0% [25]. Enhancing it with FPN improved the dice score to 81.2% using a simple SGD optimizer, which already highlights the importance of the FPN variant in considering diverse sizes of nuclei for various organs in the Kumar dataset. Adapting the framework to use an Adam optimizer [37] for faster convergence resulted in a further increase, to 82%. Introducing data augmentation techniques to consider blending inconsistencies in images due to different lighting conditions while scanning, and rotation invariance due to the rotation-invariant property of nuclei enhanced the dice score to 82.6%. Additional improvements in optimizing the Mask R-CNN framework to use OHEM [40] and focal loss [39] to boost classification accuracy resulted in a significantly high dice score accuracy of 83.1%.

**Figure 4:**
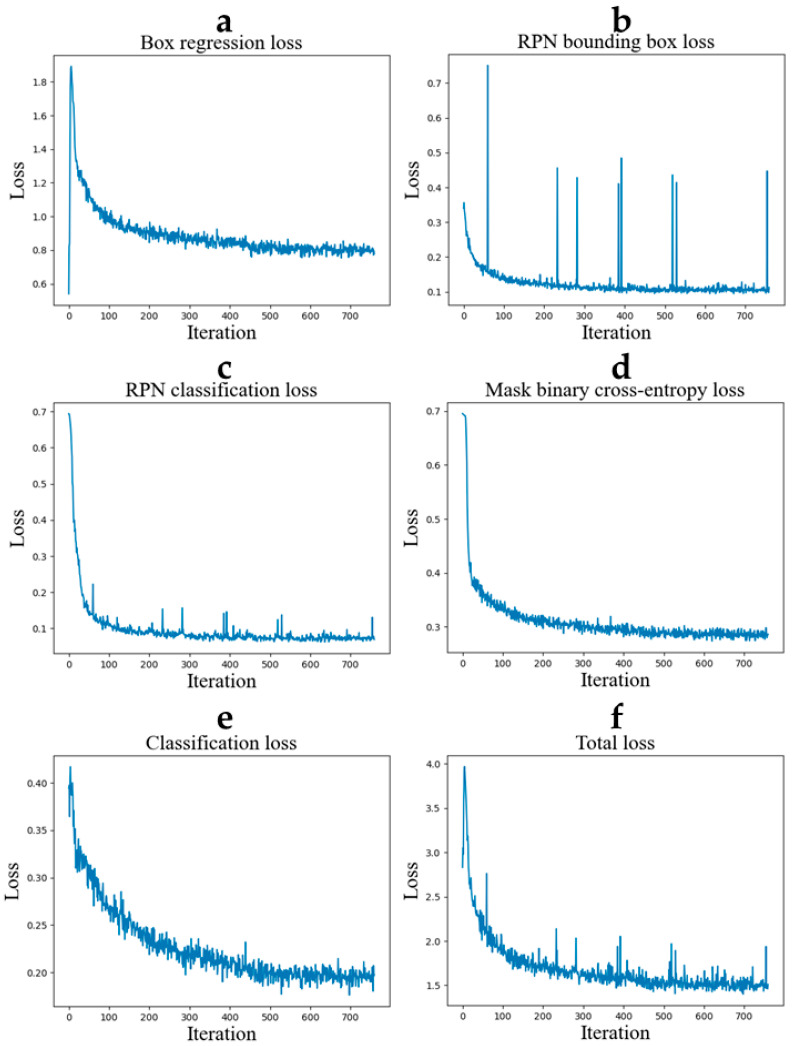
Loss convergence plots. (**a**) The box regression loss, (**b**) the RPN bounding box loss, (**c**) the RPN classification loss, (**d**) the mask binary cross-entropy loss, (**e**) the classification loss, (**f**) the total loss.

In our study, the loss convergence plots provide a visual representation of the training process and how well the model is learning over time (Figure 4). The loss convergence plots show that the training losses decrease and converge as the number of iterations increases. This indicates that the algorithm performs very well and recognizing the cell nuclei is effectively learned.

**Figure 5:**
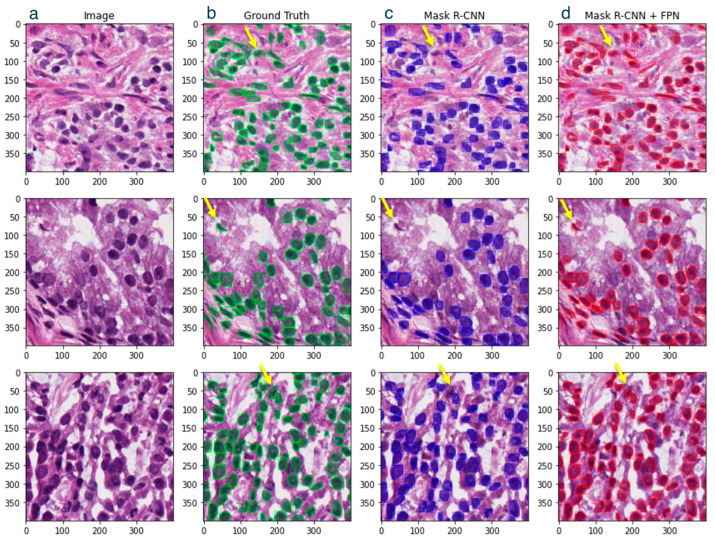
Three examples of visualizing nuclei. (**a**) Input image of H&E-stained tissue, (**b**) ground truth instance, (**c**) predicted instance using Mask R-CNN, (**d**) predicted instance using Mask R-CNN + FPN. The yellow arrows show examples of improved detection.

Based on the current statistics on the Kumar dataset, the SIS-based networks like HoVer-Net [30] and DSF-CNN [23] give an excellent dice score accuracy of 82.6% on the Kumar dataset as compared to a DIS-based network like Mask R-CNN (76%). The more recent ones like MHVN [28] and GC-MHVN [28] give even better dice scores of up to 84.3%. They combine convolution layers and attention mechanisms, which lead to even better results for the SIS-based algorithms. In this paper, we focus on improving the dice score using DIS-based networks and it can be shown here that an FPN-based Mask R-CNN gives comparable dice score accuracy of up to 83.1% (Table 3), improving the accuracy of a standard Mask R-CNN by 7.1% (Figure 5). With this progress, you can use an end-to-end trainable DIS-based algorithm and eliminate the time-consuming post-processing steps that come with SIS-based algorithms.

## 4. Discussion

So far, SIS-based networks are preferred as they are fast and simple, despite the need for traditional post-processing techniques to account for their inefficiency in separating overlapping nuclei. Moreover, even if it is known that a DIS-based network has the potential to produce better results, there is no sufficient evidence to show this on a public dataset. Here, we show that a DIS-based network, Mask R-CNN with an FPN, can also produce excellent results in identifying and detecting nuclei on histopathological images. The results observed by a DIS-based network improved from 76% to 83.1%, which is similar to the state-of-the-art results obtained by SIS-based networks (Table 3). A DIS-based approach is much more convenient for a pathologist, as it directly detects nuclei and segments them using an end-to-end pipeline, whereas an SIS-based approach forces us to deal with overlapping nuclei using traditional post-processing on segmentation masks.

A general strategy in digital pathology is to use a detection algorithm on the resulting segmentation masks of an SIS-based network [3]. One would either apply a detection algorithm like a marker-based watershed algorithm on a semantically segmented mask [43] or introduce a contour-aware network that uses a separate label to identify contours [24]. Both detection algorithms require traditional post-processing techniques to separate nuclear boundaries or to solve ambiguously classified pixels. A DIS-based network primarily focuses on localizing nuclei in the first place and later predicts a segmentation mask on the localized nuclei, which eliminates the need for such heavily engineered post-processing techniques as there is no notion of an ambiguous pixel. Therefore, a DIS-based network does not only give excellent performance on the detection and segmentation of nuclei but it is also end-to-end trainable.

From a pathological point of view, automated nuclei detection and consecutive characterization are crucial for cell counting in both tissue slides and in vitro cell constructs, such as spheroids or organoids. The detection of nuclei is the base for further steps to interpret the nucleus structure and view its malignancy. Therefore, automated detection needs to be the priority of the algorithm, rather than segmentation followed by detection.

The drawback of this approach is its computational requirements. Since a lot of research already exists for SIS-based networks, efficient networks are designed to improve the speed and performance of such networks. However, a DIS-based network, especially the Mask R-CNN, is computationally expensive as it involves two stages and spans throughout the image for various anchor sizes. Potentially, single-stage object detection approaches like YOLO [15], with an architecture based on EfficientNet [44], could be used for better performances in speed.

The resulting bounding box detections are of varied sizes after the ROI stage. It must be pooled to a constant image size to pass it into a segmentation network. Even if the ROIAlign pooling is very effective for this purpose [25], the results at the borders of nuclei could be further improved by better pooling methods. Therefore, improving pooling techniques could further improve segmentation results, thus leading to a better dice score.

Another factor that could improve dice scores is a higher accuracy in crowded regions. Nuclei detection is affected in such regions due to the use of NMS [32], which filters bounding boxes based on Intersection over Union. The detected nuclei in crowded regions could be well over the chosen NMS threshold to train the network. An alternate mechanism to filter the bounding boxes could be investigated to improve performance in such regions. The optimal detection of nuclei in crowded regions such as tumors is a challenging but highly relevant task in digital pathology.

## 5. Conclusions

Our experiments clearly demonstrated that the DIS-based Mask R-CNN, enhanced with an FPN, is a useful tool for researchers to accurately analyze and count nuclei on histopathological images. By utilizing modern training setups, such as the Adam optimizer and advanced data augmentation techniques, we achieved a dice score of 83.1%, marking a substantial improvement from the 76% accuracy of the baseline Mask R-CNN. This performance is comparable to state-of-the-art segmentation-based instance segmentation (SIS) networks, which require traditional post-processing techniques to manage overlapping nuclei. The success of the FPN variant in handling nuclei of diverse sizes and scales further underscores the potential of DIS networks in digital pathology.

However, the computational demands of DIS networks like Mask R-CNN remain a challenge. Future research should focus on optimizing these networks for better performance and speed. Single-stage object detection models like YOLO, combined with architectures like EfficientNet, could offer promising alternatives. Additionally, improving pooling techniques and addressing detection accuracy in crowded regions could further enhance segmentation results.

The approach described in this report can be used for many applications other than nuclei detection for medical research. Applications might be the detection of several types of cells in tissues, the separation of benign and malignant tissues, or even the detection of cell organelles such as mitochondria. This could be even more relevant to medical images from tissues stored in a biobank for future research and diagnostic purposes. In recent years, biobanks have grown from simple biological sample repositories to complex and dynamic units belonging to large infrastructure networks [45,46]. Such systems could benefit deeply by integrating our enhanced Mask R-CNN to detect objects in medical images. Consequently, research in digital pathology could be accelerated, and, potentially, in the future, it could be integrated into the diagnostic environment to support pathologists in analyzing histopathological images from a completely different, user-friendly perspective.

## Figures and Tables

**Table 1:** Summary of key results and methodologies of previous studies and their performance using the Kumar dataset [26].

Study	Methodology	Performance (Dice Score)	Key Findings
Kumar et al. [27]	Semantic segmentation (CNN)	76.23%	+ Achieved a moderate dice score − Struggled with overlapping nuclei
Mohta et al. [28]	MRL-based network architecture (GC-MHVN)	84.3%	+ Improved capacity, generalization, and efficiency − Pointed toward overfitting
Ronneberger et al. [29]	U-Net with post-processing	75.8%	+ Very good performance on different biomedical segmentation applications − Relied heavily on post-processing, leading to potential over-segmentation
He et al. [25]	Detection-based instance segmentation (Mask R-CNN)	76%	+ Good generalization − Computationally expensive
Graham et al. [30]	Hybrid approach (Hover-Net)	82.6%	+ Combined SIS and DIS, showing promise − Required significant computational resources

**Table 2:** Dice score with various training setups.

Network	Data Augmentation	Optimizer	Additional Hacks	Dice Score (%)
Mask R-CNN (Standard) [25]	None	SGD	None	76.0
Mask R-CNN + FPN	None	SGD	None	81.2
Mask R-CNN + FPN	None	Adam	None	82.0
Mask R-CNN + FPN	Rotation and Image-blending	Adam	None	82.6
Mask R-CNN + FPN	Rotation and Image-blending	Adam	OHEM [40] and Focal Loss [39]	83.1

**Table 3:** Dice score for various algorithms.

Type	Network	Dice Score (%)
Detection-based Instance Segmentation (DIS) Algorithms	**Mask R-CNN (ResNet50 + FPN)**	**83.1**
	Mask R-CNN (Standard) [25]	76.0
Segmentation-based Instance Segmentation (SIS) Algorithms	GC-MHVN [28]	84.3
	MHVN [28]	83.0
	DSF-Net [23]	82.6
	HoverNet [30]	82.6
	Steerable G-CNN [42]	81.8

## Data Availability

The datasets analyzed during the current study are available in the Kumar dataset, which can be accessed via their homepage, Monuseg, https://monuseg.grand-challenge.org/Data/. Download link: https://drive.google.com/file/d/1NKkSQ5T0ZNQ8aUhh0a8Dt2YKYCQXIViw/view). Also, we provide the model of this paper at the following link: https://drive.google.com/drive/folders/10jk3M94_saZoKMBfRjOkn8lCtquKk8rK (all accessed on 29 March 2023).

## Abbreviations

AI, artificial intelligence; CNN, convolutional neural networks; DIS, detection-based instance segmentation; FPN, Feature Pyramidal Network; H&E, hematoxylin and eosin; IoU, Intersection over Union; NMS, Non-Maximum Suppression; ROI, Region of Interest; RPN, Region Proposal Network; SIS, segmentation-based instance segmentation; WSI, whole-slide image.
